# Guidelines for robotic credentialling in reconstructive and functional urology. Consensus study

**DOI:** 10.1002/bco2.467

**Published:** 2024-11-20

**Authors:** Frances Harley, Rasa Ruseckaite, Eva Fong, Henry Han‐I Yao, Hashim Hashim, Helen E. O'Connell

**Affiliations:** ^1^ Department of Surgery University of Melbourne Melbourne Victoria Australia; ^2^ School of Public Health and Preventive Medicine Monash University Melbourne Victoria Australia; ^3^ Department of Urology Urology Institute Auckland New Zealand; ^4^ Department of Epidemiology and Preventive Medicine Monash University Melbourne Victoria Australia; ^5^ Bristol Urological Institute Southmead Hospital, North Bristol NHS Trust Bristol UK

**Keywords:** credentialing, curriculum, reconstructive and functional urology, robotic surgery

## Abstract

**Objectives:**

This study aims to define criteria for robotic reconstructive and functional urology credentialing using expert consensus. A recent narrative review identified a lack of standardised minimal requirements for performing robotic‐assisted surgery procedures. The substantial variability or absence of a standardised curriculum and credentialing process within a highly specialised surgical field is often insufficient to guarantee surgeon proficiency and could potentially jeopardise patient safety.

**Subjects and Methods:**

Thirty‐five international robotic surgery experts in urology and urogynaecology, selected based on surgical and research expertise, were invited to participate as expert panellists. Using a modified Delphi process the experts were asked to indicate their agreement with the proposed list of recommendations that was identified from the literature and review of relevant international credentialing policies in three electronic survey rounds.

**Results:**

Fourteen experts participated in round 1 of online surveys, 9 in round 2 and 13 in round 3. From 50 statements presented to the Delphi panel in round 1, a total of 39 recommendations (32 from round 1, 4 from round 2 and 3 from round 3) with median importance (MI) ≥ 7 and disagreement index (DI) < 1 were proposed for inclusion into the final draft set and were reviewed by the project team. Panellists agreed reconstructive and functional urology required its own specific modular training curriculum as the foundation for robotic training and a surgeon must have appropriate training i.e., fellowship or evidence of speciality training in functional urology.

**Conclusions:**

This was the first study to develop preliminary guidelines on credentialing for robotic surgery in reconstructive and functional urology. A Delphi approach was employed to establish comprehensive credentialing criteria for robotic‐assisted surgery. The consistent adoption of these criteria across institutions will foster the proficiency of robotic surgeons and has the potential to bring improvements in patient outcomes.

## 1 INTRODUCTION

Robotic‐assisted surgery (RAS) has experienced remarkable expansion since its introduction more than two decades ago. Today, it is widely used in various surgical fields. To align with this trend, healthcare networks, organisations and societies have updated their credentialing policies for robotic surgery.^1^ Many institutions have implemented basic policies related to surgeon credentialing to maintain safe and competent patient care.^2^ However, the specific qualifications for robotic sub‐specialty surgery remain variable, if non‐existent.^1^ Credentialing is a multistep process to ensure adequate clinical knowledge, robotic device familiarity and technical experience to achieve safe quality patient care and positive patient outcomes.^3^ The area of reconstructive and functional urology (RFU) has unique challenges for credentialing as there is considerable heterogeneity of procedures at a relatively low volume within this umbrella term.^4^ There are currently no universally accepted credentialing criteria for robotic surgery in RFU and individual institutions can have considerable variability in requirements.^4^ The lack of oversight associated with the adoption and use of surgical mesh and its subsequent complications highlights the importance of clinical governance during the introduction and dissemination of new procedures. Credentialing is an important part of clinical governance and is crucial in any area of novel or complex practice. Robotic surgery, particularly with the expansion of indications and new robotic procedures is a crucial area for the development of credentialing guidelines to ensure patient safety during a period of change and also with ongoing use. Our study utilised experts in robotic functional urology to develop recommendations. Credentialing of practitioners is usually done at a hospital (or hospital system) “local” level, but it is unlikely that a local credentialing committee would have the knowledge about specialised areas such as RFU, to oversee a robust process.

A detailed common framework^5^, ^6^, ^7^ for robotic curriculum includes:

1. Robotic device training. This requirement is a combination of completing a recognised robotic surgical system training course which is typically produced and managed by industry and largely based on the instructions for use, observership of cases with an experienced surgeon, simulation and wet‐lab training to develop familiarity with the system and cultivate skills.^4^ The second half of this requirement includes hands‐on sessions ideally led by experienced robotic surgeons in close partnership with trained industry experts to emphasise the safe use of the robotic surgical unit, instruments, controls and emergency procedures.^3^
2. Basic skills development: This is undertaken in several ways including lab training of wet and dry labs and simulation training. This also includes clinical training through observership and mentorship. A pre‐procedural training program in basic robotic skills could be integrated into early surgical training structures across specialties.^8^ One example of a current skills development curriculum is the Fundamentals of Robotic Surgery (FRS) training course which has been proven to be an effective program, demonstrating better performance of its trainees compared to the control group.^9^ The curricula aim to establish essential robotic surgery skills that are applicable across all subspecialties and ascertain the fundamental building blocks for procedural training as well as reinforce the theory from the prior device training. Objective measures of proficiency are an essential component of the credentialing process. These metrics can range from assessment of skills to performance errors or complications. The Global Evaluative Assessment of Robotic Skills (GEARS) is the first surgical technical skills assessment tool specifically for robotic surgery.^10^ It evaluates and scores the trainee on six domains‐ depth perception, bimanual dexterity, efficiency, autonomy, force sensitivity and robotic control.^10^ Studies that use error‐based assessment have adopted standardised definitions of task‐specific errors, such as tissue trauma, bleeding, lumen patency after anastomosis, anastomosis leakage, inversion of anastomotic margins and a positive dissection margin.^11^ Creating separate learning environments allows for focused skill acquisition, increases opportunities for robotic exposure and accelerates resident learning.^3^ During clinical training, the mentorship phase can optimally include modular training preferably within a defined curriculum which breaks down the procedure into key steps/components and entails “component surgery” advancing through the steps of increasing difficulty.^4^ This precedes advancement to undertaking the full procedure supervised by a mentor, eventually with the capacity to independently perform a procedure to a proficient standard. At this final stage, an independent proctor (who is not involved in the surgery, and reports to the credentialing committee) assesses the surgeon for sign‐off.
3. Procedural training: After completing basic skills training, trainee surgeons move on to procedure‐specific training. They learn and perform robotic procedures under the guidance of experienced instructors proficient in RAS. This training follows a modular approach, allowing trainees to progress systematically through surgical steps of increasing difficulty.^12^ Mastery of each module is necessary before advancing to the next, ensuring comprehensive competence.^13^
4. Credentialing: Recommendations for credentialing exist for some robotic procedures and are generally accepted as best practice where they have been defined by local or international societies.^1^, ^4^ However, in many areas such as RFU, there are no formal international guidelines, and this leaves credentialing in the hands of local committees who are unlikely to be subject matter experts.^4^ Multidisciplinary teams (MDTs) while a central approach to cancer care in recent years the model of MDTs has moved beyond cancer to complex benign diseases such as incontinence surgery or stone management.^14^ Although the original aim of the MDT process was a drive to increase the quality and consistency of cancer treatment; with more professionals involved, treatment decisions are less likely to reflect any one individual's view or recommended approach and instead be based on expert consensus.^15^ This approach could be vital in the RFU sub‐speciality to address skillset transferability and allow the problem of whether being signed off for one key index procedure is an adequate demonstration of proficiency to be able to do other procedures through collegiate expert consensus.

The credentialing process is based on evidence of competence. For example, certification on completed skill courses and a proctor report is required before full privileges are granted.^16^, ^17^ The challenge in developing such a framework in RFU is the relatively low case numbers in a large variety of procedures and the low numbers of subspecialised surgeons.^4^ The development of minimum caseloads will be a difficult criterion to establish with a consensus. To overcome the low numbers, it will likely require a coordinated effort to develop the simulation courses and potentially the utilisation of technology to ensure the appropriate proctorship is undertaken before credentialling. This would require the cooperation of the sub‐speciality experts to collaborate in developing this surgical approach as well as collaborating with high‐volume robotic pelvic onco‐urological surgeons. Our group recently published a narrative review highlighting the importance of developing a RAS curriculum for RFU.^4^

The aim of this project was to develop, using a Delphi consensus method, a set of recommendations establishing and clarifying credentialing for surgeons performing RFU. An international consensus amongst the leading surgeons determines an expected level of clinical experience and surgical expertise. We propose the first structured training program for RFU, intending to improve patient safety during the learning phase of the surgeon.

## 2 METHODS

### 2.1 Preliminary statements

A steering group was established following the creation and publication of a narrative review assessing the required credentials for RAS in RFU. PubMed and the grey literature were searched as part of a narrative review^4^ by the steering group for proposed general robotic surgery credentialing criteria and those considered specific to RFU. The review identified key components for a proposed robotic training programme including general requirements, i.e., surgical fellowship training, E‐knowledge, simulation training, modular training and credentialing.^4^ This review has formed the basis of the identification and refinement of the proposed Delphi statements for consensus. The statements have been collected from a review of similar studies looking at RAS curricula in surgery and personal experience. The steering group evaluated RAS credentialing policies from international institutes and societies both in urology and other specialities. A list of preliminary statements for the guidelines was based on the findings from the literature and our published narrative review. The statements were combined according to the clinical experience of the project team, revised by the project team, reworded and listed as recommendations for the review.

### 2.2 Expert recruitment

Thirty‐five experienced local and international reconstructive and functional urological surgeons, with proficiency in RAS were identified. Participants were selected based on their reputation, involvement in international panels or societies, experience with RFU, robotic surgery and contributions to the literature.

### 2.3 Data collection

Participants were initially invited electronically to complete an online survey asking to provide feedback in terms of which statements should be included in a final set of guidelines. Participants were asked to rate the validity and feasibility of the guidelines. An online classical Delphi method consisting of three survey rounds was employed in this study.^18^, ^19^ First‐round surveys were distributed using the secure Qualtrics survey software (https://www.qualtrics.com). Participants were sent regular reminder emails prompting them to complete the survey if not already done.

Invitation to the first Delphi round was sent on 25/07/2023, the second round was conducted on 14/09/2023, third round on 26/09/2023.

### 2.4 Ethics approval and consent to participate

This study was approved by the Monash University Human Research Ethics Committee (MUHREC # 37730), Melbourne, Australia. An implied consent process was utilised. The link to the surveys included an invitation to participate that explained the aims of the study, its voluntary nature, and what was required for participation. Panel member consent to participate was assumed by continuing in the first Delphi round.

### 2.5 Data analysis

The panel completed Delphi surveys using a Likert scale ranging from 1 (not important) to 9 (very important) to rank the importance of a proposed statement.^20^ The results were analysed using Excel 2013 to calculate the median importance (MI) ranging from 1 to 9 and disagreement index (DI).^20^ The DI is a continuous scale that measures the variation in expert ratings. Based on the RAND method DI of 0 represents complete agreement whereas DI ≥ 1 indicates significant disagreement or lack of consensus. If the DI exceeds 1, then the distribution meets the criteria for extreme variation in ratings.^18^, ^20^ The DI is calculated by using a standard published equation. The Delphi panel was able to refine the wording of statements for subsequent rounds and propose new ones, supported by evidence, that was felt to be important for implementation.^20^

For the first round, the panellists were able to propose procedure types or numbers as answers for a select few questions. The three questions in round 1 that did not use the Likert scale were: The index procedure for RAS functional and reconstructive surgery should be – A) Robotic Burch Colposuspension, B) Robotic retropubic sling removal, C) Robotic sacrocolpopexy, D) Robotic hysterectomy or E) other? What is the minimum number of cases in which a trainee should be mentored by an experienced trainer before they are independent surgeons? A) 10, B) 20, C) 30, D) 50 cases and What is the minimum number of cases that a trainee should be proctored by an experienced trainer before they are independent surgeons? A) 1, B) 2, C) 3 and D) 5 cases. These questions were measured as consensus was reached if 80% of panellists agreed with the same option. If 80% was not achieved, then in consensus with the steering group the questions were revised based on the group's answers and provided to the panellists in rounds 2 and 3. In Round 2, the majority of statements were revised from the statements in Round 1 that had either achieved MI > =7 or DI < =1. Three statements were identified as clinically significant by the steering group but had not been considered important or reached consensus by the panellists. These statements were subsequently revised and presented to the experts for their evaluation. Round 3 statements were revised from round 2 which did not reach a consensus.

## 3 RESULTS

### 3.1 First round

Of the 35 (10 from Australia and 25 international) experts invited to participate in this study, 18 (8 female) agreed to undertake the first online survey (round one). Fourteen were urologists and four were urogynecologists. Of these, fourteen experts completed the online survey for the first round, nine for the second and thirteen for the third round. In the initial round, participants of the Delphi panel received a comprehensive list comprising 50 recommendation statements along with a supplementary document detailing the process. Of the 50 potential statements presented to the panel in the first round, 32 (64%) statements were rated as very important (MI ≥ 7) with low disagreement (DI ≤ 1). Ten (20%) were rated with high disagreement (DI ≥ 1) and 5 statements, were considered important but had disagreement. Three statements requested panellists provide answers, and consensus at 93% was reached that the index procedure for RAS should be Robotic sacrocolpopexy. The median proposed minimum number of cases in which a trainee should be mentored by an experienced trainer before they are independent surgeons was 20 and the median proposed minimum number of proctorship cases was 3 (Appendix Table A1). After round 1, the statements rated with high disagreement were excluded from the further evaluation. The eight statements that were considered important but with high disagreement (DI ≥ 1) were re‐evaluated by the steering group, re‐written and presented to the panel with an additional two statements that were considered clinically important to the investigators but deemed unimportant by the panel.

### 3.2 Second round

The second round involved 10 statements, which had been redeveloped from round 1. Four (40%) were rated as very important (MI ≥ 7) with low disagreement (DI ≤ 1). Six remaining statements contained MI's 4–6 and a median DI of 1.46. The remaining six statements were considered relevant to key ideas on the topic so were again re‐evaluated and presented to the panel (Appendix Table A2).

### 3.3 Third round

In the third round, six statements were presented to the panel after further refinement from rejected statements in round 2. Three (50%) were rated as very important (MI ≥ 7) with low disagreement (DI ≤ 1). Of the remaining 3 statements, 2 were rated as important but DI ≥ 1. A total of 39 statements (32 first round, 4 second and 3 final rounds) with MI ≥ 7 and DI < 1 were proposed for inclusion into the final set and were reviewed by the project team (Appendix Table A3). The final set of statements was sent for review to the Delphi panel members for their feedback, which was collated in the final set (Appendix Table A4).

**FIGURE 1:**
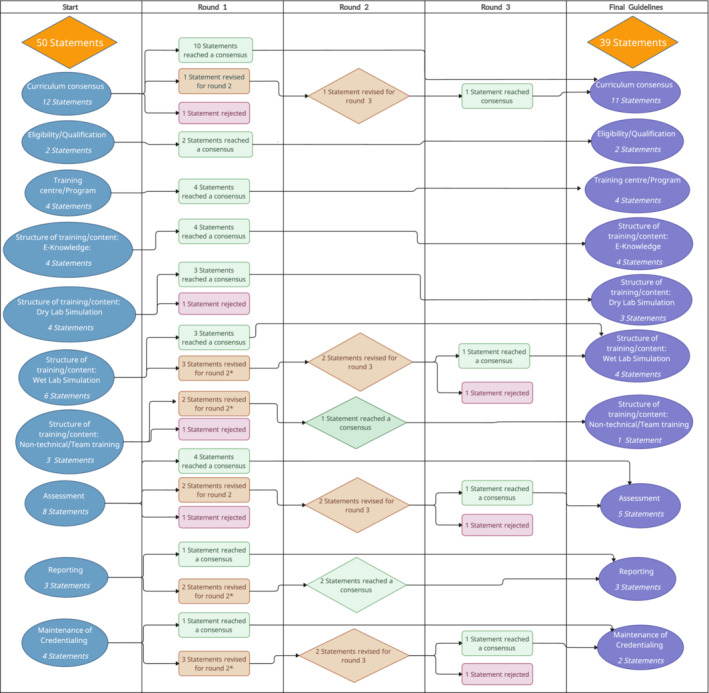
Swimlane diagram outlining the Delphi process across three rounds. *: revised statements were merged into a single statement.

A summary diagram (Figure 1) outlines the Delphi process across the three rounds.

## 4 DISCUSSION

Reconstructive and Functional urology, a subspecialty with a wide range of procedures, requires adaptability from surgeons due to challenges like distorted anatomy, re‐operative surgical fields with significant scarring or impaired tissue quality. This subspecialty exhibits an overlap between urology and urogynaecology, resulting in varying levels of experience and skill sets amongst those performing these procedures. Since the introduction of RAS to the market, concerns have persisted regarding the competence of surgeons using this evolving technology. To address these concerns and prioritise patient safety, it is essential to establish proper credentialing processes for robotic surgery. These procedures must ensure that surgeons seeking privileges in robotic surgery have acquired the necessary knowledge, and technical and nontechnical proficiencies.

Our experts reached a consensus in 39 of the proposed 66 statements across the three rounds. The experts agreed that a standardised robotic training curriculum would be advantageous for RFU and that clinical outcomes during the learning curve can be improved with adoption, this is a common opinion found across many other studies considering implementing robotic training curricula.^1^, ^6^, ^21^ Consensus was reached on the need for an agreed curriculum of prior knowledge and training in the subspeciality prior to embarking on surgery. With an expectation of medical training, appropriate professional registration and prior experience in open and/or laparoscopic surgery the specific curriculum should include E‐knowledge, simulation and bedside observation. The panellists also reached a consensus on the importance of training being tailored to the trainee's experience, whether they were robotic naïve or had prior training.

Considering the low case numbers and low surgeon numbers performing these procedures, consensus was reached by the experts on the importance of training centres being assessed and formally accredited to offer these procedures to ensure the standard of care is provided. Likewise, consensus was reached that those carrying out proctorship/mentorship should be certified, and all assessments performed should be using a standardised proforma. Consensus was reached on the minimum number of cases that an experienced trainer should mentor a trainee before they are independent surgeon is 20. Consensus could not be reached by the experts on the minimum duration of modular training or the minimum number of cases for which a surgeon should be proctored. It is commonly noted in the literature that there is a variation in cases to reach the learning curve for different RAS procedures. However, one advantage of RAS is that the learning curve is shorter^22^ compared to laparoscopy. A systematic review by Son et al.^23^ reported that approximately 50 cases were required to overcome the learning curve for laparoscopic gastrectomy, whereas only 11–25 cases were required for RAS. ROBOGYN‐1004, a large multicentre RCT reported a reduction in learning curve and operating times observed relative to a laparoscopic approach.^24^ Modular training stands as a cornerstone of contemporary surgical education. In this training framework, the final phase involves the practical application of theoretical knowledge and acquired skills to real surgical procedures. Modular training methodically deconstructs complex procedures into their fundamental steps or components. Trainees then advance through these stages, gradually tackling increased levels of complexity. This stepwise, progressive approach is an essential precursor to the transition to supervised surgical procedures in an operating room, under the guidance of a mentor. Ultimately, it equips trainees with the proficiency to independently perform procedures at a high standard. Considering the expert panellists were not able to reach a consensus on the required minimum time of mentorship or the minimum number of proctored cases, this suggests uncertainty on the expected progression of skills or expertise. It may suggest a need to study the learning curves more closely in RFU cases. It could also stem from a belief that already gained skills will be easily transferable.

Though the experts did agree to a proposed minimum case of supervised cases, which is in line with current training practices. The transition in surgical training from focusing solely on case volumes to adopting metric‐based approaches is poised to benefit RFU training significantly. This area is being propelled by advancements in technology, particularly the increasing use of virtual reality (VR) simulation and 3D hydrogel models for skills assessment, replacing traditional cadaver‐based training or supervised live training.^25^ These cutting‐edge technologies provide a highly realistic and controlled environment for practising surgical techniques, allowing for objective evaluation of skills. Moreover, they offer invaluable feedback that contributes significantly to enhancing surgical proficiency. RFU along with other RAS subspecialities should seek to leverage these advancements to address the infrequency of certain procedures. However, ongoing extensive research is needed to validate the effectiveness of these models.

A consensus was not reached on whether RAS curricula that had been developed for onco‐urology procedures such as robot‐assisted radical prostatectomy (RARP) could be used as the foundation to create the RFU curricula. After not reaching a consensus in round 2, this question was reworded by the investigation team and resubmitted to the experts where consensus was reached. The consensus is that RFU requires its own specific modular training curriculum as the foundation for RAS training since the skills are different to onco‐urology. It is assumed by the investigators that the experts believe that the early steps of robotic training such as device training and the fundamental skills apply to all robotic specialities and the difference in training needed for RFU is to be focused on its index procedures and not relying on transferable skills. Device training and foundation skills are considered essential introductory skills applicable to all robotic surgeons, regardless of their specialty. These basic skills are crucial before proceeding to procedure‐specific training like RARP. This opinion by the experts was interesting as the majority of surgical training particularly for RAS is undertaken in onco‐urology with trainees and surgeons having a higher volume of procedures and more operative hours performing these cases. It was thought by the investigating group that skills and techniques developed from onco‐urology would at least be partially transferrable to RFU procedures and RARP could be considered an index procedure for all urologists starting robotic training. However, the consensus from the experts is that the skillset required to undertake RFU is separate and necessitates its own intensive training. The experts may consider certain aspects of RARP training to not apply to all types of robotic surgery. When distinguishing between foundational skills and procedure‐specific skills in robotic surgery training, it's crucial to consider the limitations of time‐based assessments as a measure of proficiency. Merely attending a training session should not be equated with acquiring proficiency; active hands‐on training is essential for assessing surgical skills accurately. Therefore, a critical evaluation is necessary to determine the effectiveness of training methods in measuring surgical proficiency regardless of the speciality.

Outcomes in RFU typically include functional and patient‐reported outcomes, as opposed to biochemical or radiological in onco‐urology. In round 1 the experts came to the consensus that RFU surgeons should have to enquire and report on specific outcomes such as bowel, bladder, ureteric, vascular injury, fistula rates and chronic pain. These outcomes were thought by the investigating team to be more representative of “successful” surgical outcomes than those typically reported outcomes such as blood loss, operative time or length of stay alone. These outcomes though rare, appropriately reflect the possible challenges encountered during these procedures and would serve as a good indicator of surgeon proficiency along with patient‐reported outcome measures (PROMs).^26^ Specific reporting would facilitate outcome comparison with peers, assisting with identifying variations in technique and patient care and targeting possible areas for improvement.^27^ The challenge that RFU poses is that of infrequent cases with heterogeneity of case mix. The typical measure for robotic proficiency has been case numbers due to the assumed ability to reach and maintain proficiency in the context of robotic onco‐urology.^28^, ^29^

An area that the expertise did not consider very important was the maintenance of credentialing. The experts rejected three of the five proposed statements concerning guidelines of possible criteria to maintain credentialing. They did not reach a consensus in the third round on ‘Surgeons should have to undertake credentialing maintenance with hospitals or societies with ongoing review of cases (video recording, outcomes such as PROMs, blood loss etc)’ and that ‘Credentials should be transferable between institutions and countries. The concepts that were rejected in the first round included the need for an annual review of outcomes to main credentials and that surgeons should undergo procedure‐specific credentialing if they want to start undertaking a new procedure in an institution. Though not entirely clear why these concepts were rejected it could be presumed that though surgeons agree patient safety is paramount, there would be an increased administrative load placed on the surgeons to partake in this regular maintenance of credentialing. This avoidance of adding to mental load, particularly as many surgeons operate at multiple hospitals/health services may be why the experts favoured the idea that credentialling gained should be universal and therefore transferable. Another factor that affects the maintenance of credentialling is the low case numbers in RFU, this could potentially be mitigated with the inclusion simulation. However again this must be balanced against the burden of time simulation would place on surgeons amongst other activities and the range of procedures which cannot all be simulated from a practical standpoint.

Lastly, consensus was reached in a couple of statements regarding credentialing and maintenance of credentialing. Credentialing by healthcare organisations is the process used to verify the qualifications and experience of a clinician to determine their ability to provide safe, high‐quality healthcare within a specific healthcare setting and role.^30^ Credentialing holds the potential to enhance patient safety by guaranteeing that healthcare providers operate within the confines of their training (often signalled by logbook and specific qualifications) competence and within the capacity of the service in which they are working.^30^ The consensus was also reached by the experts that surgeons should have to undertake credentialing maintenance with hospitals or societies with ongoing review of cases through proctored observation or video recordings and reporting of PROMs and routine operative outcomes such as length of stay and blood loss. Consensus was also reached that credentialling should be transferable between institutions and countries. The agreement that credentialing should be transferable reassigns accountability for a surgeon's training and expertise to surgical colleges and societies, as opposed to hospitals. Whether hospitals, colleges and societies are equipped to do this, particularly assessment of skill maintenance, is another complex problem.

### 4.1 Strengths and limitations/challenges

This was the first study to develop guidelines for RFU robotic surgery curriculum using a Delphi consensus process. The primary strength of this study is the status of international recruitment, with all participants being global leaders in this field. It suggests this study's recommendations will be applicable across health services and countries and is a true representative opinion of the current leading experts in the field on the essential components necessary to achieve and maintain baseline requirements of training. Though there are no strict requirements for a Delphi study sample size, a relatively small panel size was the primary limitation of our study. A minimum sample size of 10 is usually intended to acquire adequate information and make valid conclusions about the research study.^31^ It is not uncommon for a small number of professional panels of similarly trained experts to provide and develop reliable criteria and recommendations that maintain valuable decision‐making. Our study had an unexplained drop in response to the Round 2 survey compared to one and three which may have an impact on the consensus reached and the statements provided in Round 3, however since there were only 10 statements in that round it is thought the impact will be minimal.

## 5 CONCLUSION

This modified Delphi study created a set of recommendations (appendix Table A1, A4) establishing and clarifying credentialing for robotic surgeons performing RFU by international expert robotic surgeons using a systematic approach. Developing preliminary recommendations is an important first step, as is supporting standardised surgical training and advocating for minimal standards of surgeon proficiency. Implementation of these criteria uniformly across institutions may ensure the proficiency of robotic surgeons and has the potential to positively impact patient outcomes.

## AUTHOR CONTRIBUTIONS

Eva Fong, Henry Han‐I Yao, Hashim Hashim and Helen O'Connell created the initial concept of the work. Frances Harley wrote the initial manuscript. Rasa Ruseckaite performed the statistical analyses. All authors refined the final manuscript and agreed to be accountable for all aspects of the work.

## CONFLICT OF INTEREST STATEMENT

The authors declare that they have no disclosure of interest.

## ETHICAL STANDARDS INCLUDING INFORMED CONSENT

This article does not contain any studies with human participants or animals performed by any of the authors.

## APPENDIX A

**TABLE A1:** Round 1 Delphi study results.

Round 1 result‐ responses with MI > = 7 and DI < =1	Median importance (MI)	Disagreement index (DI)
**Curriculum consensus**		
Do you agree that a standardised robotic training curriculum for reconstructive and functional urology will be advantageous to robotic training?	8.1	0.27
Clinical outcomes during RAS learning curve can be improved by the adoption of a standardised curriculum for training?	8	0.41
Robotic curriculum training should be tailored to the experience of the different target groups to include fellow with some robotic experience?	8	0.29
Robotic curriculum training should be tailored to the experience of the different target groups to include surgeon in practice with no previous robotic experience (excepting assisting)?	8	0.43
Robotic curriculum training should be tailored to the experience of the different target groups to include surgeon with fellowship training in robotic surgery?	8	0.43
The underpinning theoretical (basic) reconstructive urology robotic curriculum should include Baseline evaluation?	8	0.29
The basic robotic curriculum should include E‐learning module (online access to information)?	8	0.54
The basic robotic curriculum should include simulation‐based training?	8	0.64
The basic robotic curriculum should include Robotic theatre (bedside observation)?	8	0.24
The basic robotic curriculum should include all the above?	8	0.54
**Eligibility/Qualification**		
A surgeon must have appropriate training ie fellowship OR evidence of training in functional urology in subspeciality including comprehensive knowledge of the conditions being treated?	9	0.41
Before beginning the curriculum, the candidate should have a minimum tableside assistance experience of 10 RAS cases?	8	0.64
**Training centre/Program**		
Training centres should be assessed and accredited via a recognised training agency/society?	7	0.60
Training centres accredited should be related to case volume in the specialty via a recognised agency/society?	7	0.83
Do you think case volume should be considered in the accreditation of a training centre?	7	0.53
Formal assessment should occur at completion of each component of training?	7	0.65
**Structure of training/content: E‐Knowledge**		
Training must include assessment of knowledge of a robotic system and of operative room setup?	8	0.41
Training must include completion of the online robotic system theoretical training package?	8	0.60
Assessment must test awareness of the fundamentals of the robotic system components and instrumentation?	8	0.41
Each section of the e‐learning should have questions to evaluate knowledge?	8	0.41
**Structure of training/content: Dry Lab Simulation**		
Assessment should include demonstrating competency of operation set‐up, docking of robotic system, port placement and instrument use?	8	0.49
Trainees need to demonstrate competency in manipulation of instruments to achieve key competency tasks awareness of ports and instruments, haemostasis, suturing, swapping instruments, swapping arms.	8	0.49
Assessment should also include negative such as loss of arm awareness/not visualised and injury to the animal off‐screen.	8	0.49
**Structure of training/content: Wet Lab Simulation**		
Each procedure should be divided into key steps and to be able to proceed, must complete each step to a satisfactory level	8	0.49
Progression of the trainee through the different steps must follow a modular pattern according to the level of complexity of each step?	8	0.49
The assessment of each step of modular training should be monitored by experienced/credentialed surgeon? The wet lab training can be monitored by an experienced evaluator.	8	0.64
**Assessment**		
The index procedure for RAS functional and reconstructive surgery should be Robotic Burch Colposuspension Robotic retropubic sling removal Robotic sacrocolpopexy Robotic hysterectomy	Robotic sacrocolpopexy	N/A
Trainers/proctors should be assessed and certified?	8	0.97
There should be a standardised form for those providing mentorship?	8	0.64
There should be standardised proctor assessment form specific to reconstructive and functional urologists/urogynaecologists?	8	0.64
**Reporting**		
Specific functional and reconstructive urology outcomes should be included in reporting such as as bowel, bladder, ureteric, vascular injury, fistula rates and chronic pain?	8	0.43
**Maintenance of Credentialing**		
A surgeon's credentialing should be transferable between institutions and countries?	8	0.43
Round 1 result‐ Responses with MI > = 7 and/or DI > 1 that were revised for Round 2		
**Curriculum consensus**		
The established structure of onco‐urological procedures curriculum such as RARP should be applied to the RFU curriculum?	7	0.91
**Structure of training/content: Dry Lab Simulation**		
Assessment should include demonstrating port placement, instrument use and entry into peritoneum?	9	1.01
**Structure of training/content: Wet Lab Simulation**		
Assessment should include demonstrating competency of operation set up, docking of robotic system, port placement and instrument use?	8	1.94
**Structure of training/content: Nontechnical/Team training**		
Nontechnical skills training should include decision‐making and emergency scenarios?	8	1.09
**Assessment**		
What is the minimum number of cases that a trainee should be mentored by an experienced trainer before they are independent surgeons? 10 20 30 50	20	NA
What is the minimum number of cases that a trainee should be proctored by an experienced trainer before they are independent surgeons? 1 2 3 5	3	NA
**Reporting**		
Surgeons should report their outcomes and patient‐reported outcomes as part of their assessment?	7	0.46
Surgeons should continue to report their outcomes and patient‐reported outcomes after ‘certification’ with a standardised reporting template?	7	0.65
Round 1 result‐ Responses with MI < = 7 and DI > =1		
**Curriculum consensus**		
Robotic curriculum training should be tailored to the experience of the different target groups to include Trainee/fellow with no previous robotic experience?	7	1.68
**Structure of training/content: Wet Lab Simulation**		
Assessment should include on animal models demonstrating port placement, instrument use and entry into peritoneum?	5	2.00
The duration of the modular training activity should be 6 or 12 months?	5	1.01
**Structure of training/content: Nontechnical/Team training**		
Nontechnical skills training and operative team training should be included as part of the assessment?	7	1.56
Nontechnical skills can be sufficiently assessed with NOTSS (NonTechnical Skills for Surgeons)?	6	1.36
**Assessment**		
There should be formal assessment of each stage of training.	6.5	0.64
The final assessment should be based on the evaluation of an index video by certified independent examiners in a blind review process.	6	1.98
**Maintenance of Credentialing**		
Surgeons should undergo annual review of cases to maintain credentialing?	6	2.21
Surgeons should report as part of their annual review complications/adverse events and case outcomes both objective and patient reported outcomes?	7	1.23
A surgeon should undergo procedure‐specific credentialing if wanting to start undertaking a new procedure in an institution?	6	1.44

**TABLE A2:** Round 2 Delphi study results.

Round 2 results	Median importance (MI)	Disagreement index (DI)
Nontechnical skills training and operative team training such as decision‐making and emergency scenarios should be included in the training?	8	0.54
The minimum number of cases that an experienced trainer should mentor a trainee before they are independent surgeon is 20?	8	0.33
Outcomes such as complications, blood loss, length of stay and patient‐reported outcomes should be reported by the surgeon as part of their assessment?	7	0.59
Surgeons should keep records and continue to report on their outcomes using a standardised template after completing their training?	7	0.67
The minimum number of cases that an experienced trainer should proctor a trainee before they are independent surgeons is 3?	5	3.4
Surgeons should have ongoing reviews of cases as part of credentialing maintenance?	6	0.3
Should the already established uro‐oncology (RARP) modular training curriculum be the foundation for RAS training?	5	1.26
Assessment should include demonstrating competency in a theatre setup, docking, port placement and instrument use in wet lab/cadaver or animal models?	4	2.72
The duration of the modular training activity should be 6 months?	5	1.68
Surgeons should have to prove specific procedure training as part of credentialing if wanting to undertake a new procedure?	6	1.24

**TABLE A3:** Round 3 Delphi study results.

Round 3 results	Median importance (MI)	Disagreement index (DI)
Reconstructive and functional urology requires its own specific modular training curriculum as the foundation for RAS training, as the skills are different to uro‐oncology	8	0.75
Part of simulation training should include assessing competency at positioning, docking, port placement and instrument use in models?	8	0.69
Surgeons should have to undertake credentialing maintenance with hospitals or societies with ongoing review of cases (video recording, outcomes such as PROMs, blood loss, etc.)	7	0.47
The minimum number of cases that a trainee should be proctored by an experienced trainer before they are independent surgeons is 5?	7	2.09
The minimum duration of the modular training activity should be 6 months?	5	1.58
If surgeons want to start performing a new procedure, they should have to prove prior training and/or experience as part of credentialing.	7	1.45

**TABLE A4:** Final set of guidelines for credentialing in reconstructive and functional urology.

Final guidelines	Median importance (MI)	Disagreement index (DI)
**Curriculum consensus**		
1. Do you agree that a standardised robotic training curriculum for reconstructive and functional urology will be advantageous to robotic training?	8.1	0.27
2. Reconstructive and functional urology requires its own specific modular training curriculum as the foundation for RAS training, as the skills are different to uro‐oncology	8	0.75
3. Clinical outcomes during the RAS learning curve can be improved by the adoption of a standardised curriculum for training.	8	0.41
4. Robotic curriculum training should be tailored to the experience of the different target groups to include surgeon in practice with no previous robotic experience (excepting assisting)?	8	0.43
5. Robotic curriculum training should be tailored to the experience of the different target groups to include surgeon with fellowship training in robotic surgery?	8	0.43
6. The underpinning theoretical (basic) reconstructive urology robotic curriculum should include Baseline evaluation?	8	0.29
7.The basic robotic curriculum should include El‐earning module (online access to information)?	8	0.54
8. The basic robotic curriculum should include simulation‐based training?	8	0.64
9. The basic robotic curriculum should include Robotic theatre (bedside observation)?	8	0.24
10. The basic robotic curriculum should include all the above?	8	0.54
**Eligibility/Qualification**		
11. A surgeon must have appropriate training ie fellowship OR evidence of training in functional urology in subspeciality including comprehensive knowledge of the conditions being treated?	9	0.41
12. Before beginning the curriculum, the candidate should have a minimum tableside assistance experience of 10 RAS cases?	8	0.64
**Training centre/Program**		
13. Training centres should be assessed and accredited via a recognised training agency/society?	7	0.60
14. Training centres accredited should be related to case volume in the specialty via a recognised agency/society?	7	0.83
15.Do you think case volume should be considered in the accreditation of a training centre?	7	0.53
16. Formal assessment should occur at completion of each component of training?	7	0.65
**Structure of training/content: E‐Knowledge**		
17. Training must include assessment of knowledge of a robotic system and of operative room setup?	8	0.41
18. Training must include completion of the online robotic system theoretical training package?	8	0.60
19. Assessment must test awareness of the fundamentals of the robotic system components and instrumentation?	8	0.41
20. Each section of the e‐learning should have questions to evaluate knowledge?	8	0.41
**Structure of training/content: Dry Lab Simulation**		
21. Assessment should include demonstrating competency of operation set up, docking of robotic system, port placement and instrument use?	8	0.49
22. Trainees need to demonstrate competency in manipulation of instruments to achieve key competency tasks awareness of ports and instruments, haemostasis, suturing, swapping instruments, swapping arms.	8	0.49
23. Assessment should also include negative such as loss of arm awareness/not visualised and injury to the animal off screen.	8	0.49
**Structure of training/content: Wet Lab Simulation**		
24. Each procedure should be divided into key steps and to be able to proceed, must complete each step to a satisfactory level	8	0.49
25. Progression of the trainee through the different steps must follow a modular pattern according to the level of complexity of each step?	8	0.49
26.The assessment of each step of modular training should be monitored by experienced/credentialed surgeon? The wet lab training can be monitored by an experienced evaluator.	8	0.64
27. Part of simulation training should include assessing competency at positioning, docking, port placement and instrument use in models?	8	0.69
**Structure of training/content: Non‐technical/Team training**		
28. Non‐technical skills training and operative team training such as decision‐making and emergency scenarios should be included in the training?	8	0.54
**Assessment**		
29. The index procedure for RAS functional and reconstructive surgery should be?	Robotic sacrocolpopexy	N/A
30. Trainers/proctors should be assessed and certified?	8	0.97
31. There should be a standardised form for those providing mentorship?	8	0.64
32. There should be standardised proctor assessment form specific to reconstructive and functional urologists/urogynaecologists?	8	0.64
33. The minimum number of cases that an experienced trainer should mentor a trainee before they are independent surgeon is 20?	8	0.33
**Reporting**		
34. Outcomes such as complications, blood loss, length of stay and patient‐reported outcomes should be reported by the surgeon as part of their assessment?	7	0.59
35. Surgeons should keep records and continue to report on their outcomes using a standardised template after completing their training?	7	0.67
36. Specific functional and reconstructive urology outcomes should be included in reporting such as bowel, bladder, ureteric, vascular injury, fistula rates and chronic pain?	8	0.43
**Maintenance of Credentialing**		
37.Surgeons should have to undertake credentialing maintenance with hospitals or societies with ongoing review of cases (video recording, outcomes such as PROMs, blood loss, etc.)	7	0.47
38.A surgeons credentialing should be transferable between institutions and countries?	8	0.43
